# Comparative effectiveness of a serious game and an e-module to support patient safety knowledge and awareness

**DOI:** 10.1186/s12909-016-0836-5

**Published:** 2017-02-02

**Authors:** Mary E. W. Dankbaar, Olivier Richters, Cor J. Kalkman, Gerrie Prins, Olle T. J. ten Cate, Jeroen J. G. van Merrienboer, Stephanie C. E. Schuit

**Affiliations:** 1 Institute of Medical Education Research, Erasmus University Medical Center, PO Box 2040, 3000 CA Rotterdam, The Netherlands; 2 Faculty of Business Informatics, University of Utrecht, Utrecht, The Netherlands; 3 Department of Anesthesiology at University Medical Center Utrecht, Utrecht, The Netherlands; 4 Department of Internal Medicine, Erasmus University Medical Center, Rotterdam, The Netherlands; 5 Center for Research and Development of Education, University Medical Center Utrecht, Utrecht, The Netherlands; 6 Department of Health, Medicine and Life Sciences, Educational Development and Research, Maastricht University, Maastricht, The Netherlands; 7 Department of Emergency Care and Internal Medicine, Erasmus University Medical Center, Rotterdam, The Netherlands

**Keywords:** Serious games, Motivation, Knowledge, Performance, Self-efficacy, Design-based research, Patient Safety

## Abstract

**Background:**

Serious games have the potential to teach complex cognitive skills in an engaging way, at relatively low costs. Their flexibility in use and scalability makes them an attractive learning tool, but more research is needed on the effectiveness of serious games compared to more traditional formats such e-modules. We investigated whether undergraduate medical students developed better knowledge and awareness and were more motivated after learning about patient-safety through a serious game than peers who studied the same topics using an e-module.

**Methods:**

Fourth-year medical students were randomly assigned to either a serious game that included video-lectures, biofeedback exercises and patient missions (*n* = 32) or an e-module, that included text-based lectures on the same topics (*n* = 34). A third group acted as a historical control-group without extra education (*n* = 37). After the intervention, which took place during the clinical introduction course, before the start of the first rotation, all students completed a knowledge test, a self-efficacy test and a motivation questionnaire. During the following 10-week clinical rotation they filled out weekly questionnaires on patient-safety awareness and stress.

**Results:**

The results showed patient safety knowledge had equally improved in the game group and e-module group compared to controls, who received no extra education. Average learning-time was 3 h for the game and 1 h for the e-module-group. The serious game was evaluated as more engaging; the e-module as more easy to use. During rotations, students in the three groups reported low and similar levels of patient-safety awareness and stress. Students who had treated patients successfully during game missions experienced higher self-efficacy and less stress during their rotation than students who treated patients unsuccessfully.

**Conclusions:**

Video-lectures (in a game) and text-based lectures (in an e-module) can be equally effective in developing knowledge on specific topics. Although serious games are strongly engaging for students and stimulate them to study longer, they do not necessarily result in better performance in patient safety issues.

**Electronic supplementary material:**

The online version of this article (doi:10.1186/s12909-016-0836-5) contains supplementary material, which is available to authorized users.

## Background

Online instructional formats such as e-modules and computer-based simulation programs are known to enhance knowledge [1–3] and skills [4, 5] and offer flexible and cost-effective learning opportunities [6]. Serious games offer new experiential, engaging learning opportunities for complex skills learning [7]. Generally, games provide meaningful and challenging tasks, where learners directly experience the consequences of their decisions. The rationale for putting tasks at the basis of a learning environment is to promote application of knowledge and skills and transfer to practice [8]. Serious games are developed for a variety of learning goals, such as crisis management, managing disease outbreaks, ethics training or patient care. Effectiveness studies on serious games however have shown mixed and ambiguous results [9–12]. Examples of such flaws are: use of pre-to-post comparisons, leading to an upward bias of the game-effect; internal validity threats, such as history and selection [10] and a lack of studies with a suitable control condition or RCT [9]. This implies the effectiveness of serious games for learning is not clear.

Important determinants of patient safety in hospitals are awareness of safety risks by junior doctors, in addition to the skills of these doctors and their supervisors. Junior doctors are typically first responders to deteriorating patients during nights and weekends; lack of patient safety training for clinicians appears to be a major contributor to preventable harm [13]. Improving safety is not just about enhancing knowledge or skills, but also concerns the addressing of human factors and poor performance of non-technical skills that can lead to error [14]. The aviation industry previously recognized that many major incidents were the result of failures in non-technical skills [15] and have now incorporated team training and situational awareness in their training programs, but the introduction of such training in healthcare has been slow in most countries [16, 17]. Considering the tight training budgets and limited training time in health care organizations, novel and more efficient training formats are needed [2]. Games in patient safety education have the potential to teach awareness and the basics of teamwork skills that are typically acquired in simulation settings, but at a fraction of the costs [18]. In a pilot study, medical students’ knowledge on patient safety and self-efficacy was improved after playing a serious game on patient safety (Air-Medic Sky-1); they also perceived it as an entertaining format [19]. In the current study, our aim was to compare the effects of this game with an e-module for knowledge, patient safety awareness and motivation. If serious games prove to be engaging as well as effective for learners, their flexibility in use and scalability offer important advantages over traditional formats. An online game is scalable as it can, once developed, teach large groups of trainees with no extra costs per person.

In the present study, we investigated whether undergraduate medical students developed better patient safety knowledge and awareness (during their rotation) and were more motivated after engaging with a serious game than after studying a simple e-module. Both groups were compared with a historic control group who participated 6 months before the intervention groups in the study and received no additional education on patient safety. The game included video-lectures on patient safety issues, biofeedback exercises for stress management and patient-missions to stimulate patient safety awareness. The e-module was text-based, with (as far as possible, considering the format) the same content. As it was not possible to develop comparable content in the e-module for all game components (e.g. for the patient missions this was not possible), we described the learning goals, we provide an overview of the components of both formats, with their learning goals and the way learning was assessed (Table 1). It is clear from this table that there was little overlap in goals between the components, which made it easier to draw conclusions on learning effects. Following Kirkpatrick’s framework of evaluation [20, 21] we assessed students’ satisfaction with the game and e-module (level 1), their knowledge and self-efficacy (level 2), and their self-reported stress and patient safety awareness during practice (between level 2 and 3). Our hypotheses were:

1. knowledge on patient safety will improve among students in both the game and e-module group, compared to the control group, as video and text-based lectures are both hypothesized to have a positive effect on knowledge.
2. self-efficacy and patient safety awareness will be higher in the game group than in both other groups, as there are no real substitutes for the game-based missions in the e-module.
3. Perceived stress in subsequent clinical rotations will be lower in the game group than in the e-module or control group, as biofeedback exercises are hypothesized to provide more adequate feedback than text-based exercises or nothing.
4. Students in the game group will be more motivated to learn with the game than students from the e-module group, as games are expected to lead to engaged and motivated learning [9].

**Table 1:** Overview of how the two intervention programs cover the learning goals and assessment

Learning goals	Serious game	E-module	Assessment instrument
Knowledge on patient safety issues	Video lectures	Written text on the same content	Knowledge test (MC questions)
Stress management	Biofeedback exercises	Written text on biofeedback exercises	- Self-efficacy/stress management - (Self-reported) stress management during clinical rotation
- Stimulate patient safety awareness - Learn to perform effective teamwork & communication	Patient missions: diagnosing and treating virtual patients, while communicating and collaborating optimally with nurses, supervisors and the patient’s family	-	- Self-efficacy/communication - (Self-reported) patient safety behaviour during clinical rotation: awareness of adverse events and actions undertaken

## Methods

### Setting, design and selection of participants

The research population consisted of fourth-year undergraduate medical students, doing a clinical introduction course immediately before the start of a 10-week Internal Medicine rotation (first rotation). The one-week introduction course consisted of clinical training and patient safety education.

Students who consented to participate from September 2013-February 2014 were randomly assigned to a game group and an e-module group (using the Excel random function). Students who did the introduction course 6 months earlier, from April–September 2013, and consented to participate were used as historical controls. This design was used to prevent the risk of contamination of conditions as students from the e-module or game group might share access with the control group (many 4^th^year-medical students know each other well).

Primary outcome measures were: (1) knowledge on patient safety, (2) self-efficacy in patient safety issues and (3) motivation to use the e-module or game. In addition (4) self-reported stress and patient safety awareness during the clinical rotation were assessed.

On the first day of the introduction course, students were asked to participate in the study. When students from the control group consented, they received no extra instruction. When students from the game/e-module group consented, they received the game (including the finger sensors) or a link to the e-module on the same day, with a brief instruction. Students used the game or e-module at home, at a moment of their choice, using their own PC. They were free to access it several times. On the last day of the course, all groups completed a knowledge test, a self-efficacy test and an evaluation questionnaire. Students from the game group returned the game and game-data were extracted. During the following 10-week clinical rotation, they filled out a weekly questionnaire on perceived stress and patient safety issues. Every 2 weeks, approximately 13 students started this introduction course and the following clerkship Internal Medicine. In general fourth-year medical students from our institution are 23–24 years old, and two third is female. An iPad was raffled among participating students and continued recruitment until groups consisted of 40 students. Data collection took 12 months.

### Participants

Of 52 eligible students 39 consented to participate and were assigned to the control group (75%). Of 156 eligible students, 90 consented to participate in the e-module/game group (58%), they were randomly allocated to either the game or e-module group. Students with empty or incomplete surveys (less than 2 questionnaires, usually because they reported that they could not find time to do the game or e-module) were eliminated from the study, leaving 37 students in the control group (54% female), 34 in the e-module (68% female) and 32 in the game group (78% female). In total 103 students participated, with no differences between groups in GPA bachelor (average GPA of control group was 6.5 (SD = 0.47), of e-module group 6.8 (SD = 0.52) and of game group 6.7 (SD = 0.32; F = 2.8 (2), *p* = 0.07) or in gender (x^2^ (2) = 4.49, *p* = 0.11).

### Materials

#### Serious game ‘Air Medic Sky-1’

The serious game called Air-Medic-Sky-1 on patient safety was developed by the Patient Safety Center from the University Medical Center Utrecht, the Netherlands for starting residents and medical students. Learning goals of the game are to stimulate patient safety awareness (safe communication and teamwork in clinical situations) and personal stress management (practiced during relaxation exercises) [19]. The game consists of three parts (Table 1 provides an overview): (1) Brief video lectures by international experts about patient safety topics such as communication, focus under stress, teamwork. The lectures provide an important knowledge base for the patient care missions in the game. Players gain points by watching the videos, as they provide an important knowledge base (by explaining central issues of patient safety) for the rest of the game. (2) Biofeedback-breathing exercises to enable learners to consciously influence their own stress levels; the game comes with a biofeedback device with finger sensors to measure heart rate variability and skin conductance. Players gain points by watching video lectures and by doing breathing exercises (e.g. after physical relaxation, shown by a lower heart rate and lower skin conductance, the player is able to execute certain tasks in the game and will gain points). After gathering a certain amount of points, the player will be invited to participate in the patient missions. (3) Patient care missions to disaster areas around the world in a virtual flying hospital, where patients can be diagnosed and treated. During these missions, players learn to combine treating several patients simultaneously, while communicating and collaborating with superiors and nurses; they gain patient-care points by doing this successfully. The game teaches learners about patient safety, while also engaging them in exercises to control their physiology, using game play and biofeedback (see www.airmedicsky1.org for a brief explanation and demo). The three parts of the game are considered to combine important aspects of patient safety.

**Fig. 1:**
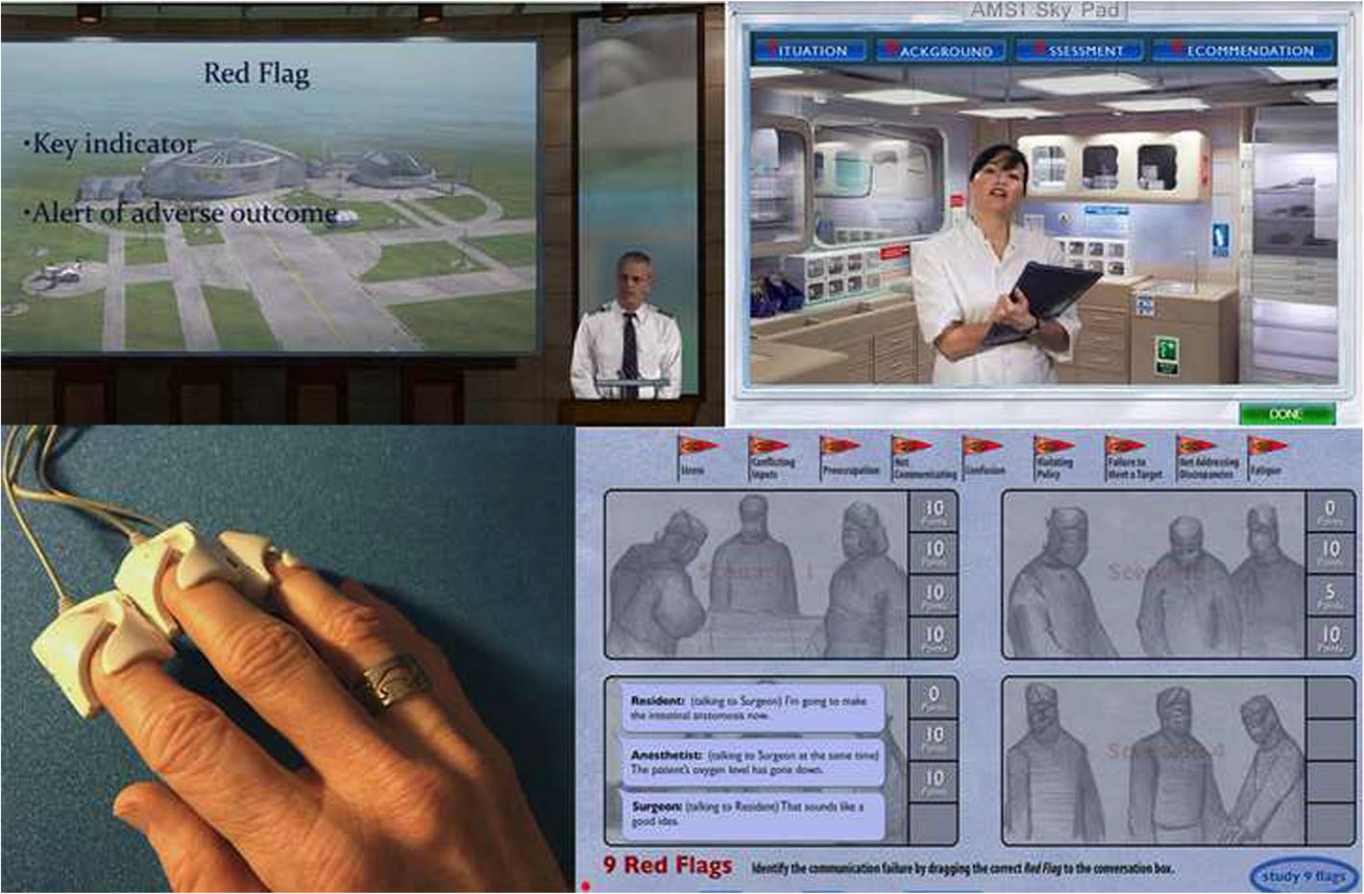
Three screenshots from the Air Medic Sky-1 Game and a photo of the finger sensors

From previous beta testing it was clear that students need 3 to 4 h to play all main sections of the game. The game was played from a USB device that was to be handed in afterwards (see Fig. 1 for game screenshots). Students could skip or replay parts of the game as desired. Game activities were logged; activities for the e-module could not be logged.

#### E-module

**Fig. 2:**
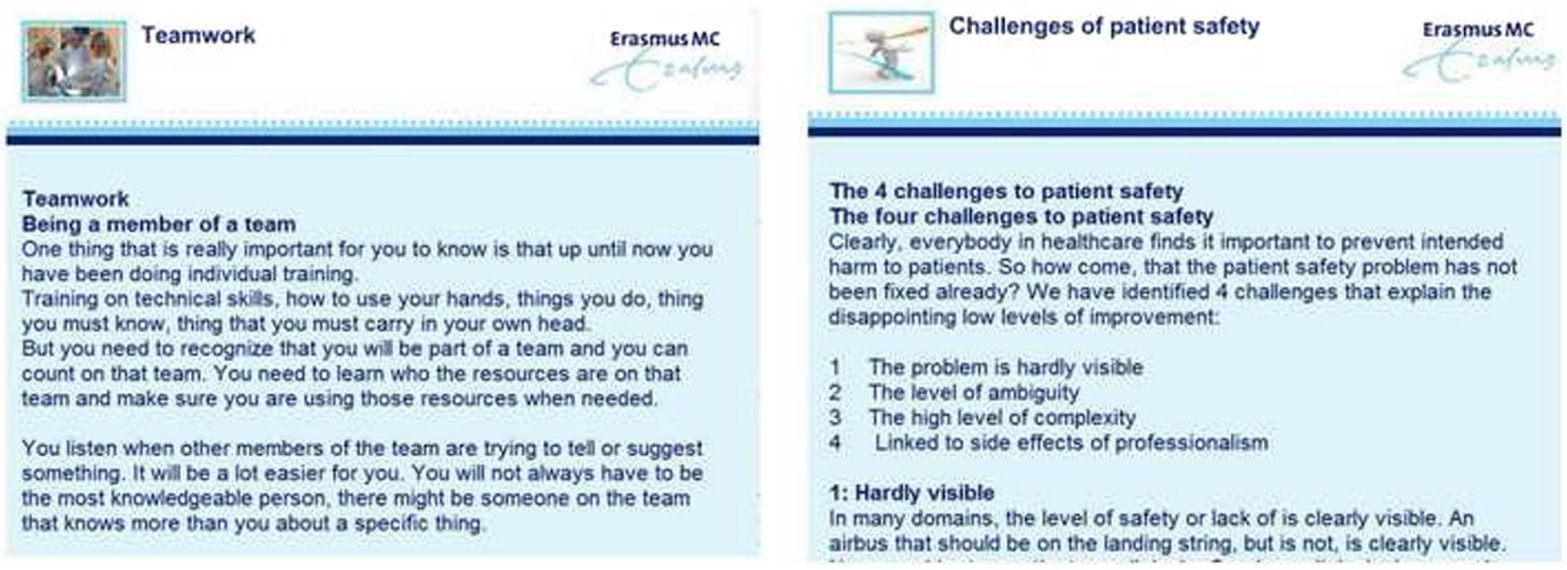
Screenshots from the E-module

For this study we developed an e-learning module on patient safety, covering the topics from the video lectures (communication, focus under stress, teamwork, etc.) and stress management. The information on patient safety was offered as structured, written text, with limited interaction. Breathing exercises were also provided in text. No replacement for the patient missions was provided, as interactively treating patients was not possible in this format. We estimated that students needed 1–2 h to study the e-module (Fig. 2 shows screenshots). Table 1 provides an overview of the two experimental conditions including learning goals and assessment instruments.

### Assessment instruments and questionnaire

#### Knowledge test

The knowledge test consisted of 70 multiple choice (MC) questions; true/false questions, four or five answer MC questions and 8 multiple answer questions (max 97 points). The knowledge test was designed by patient safety and education experts at University Medical Center Utrecht and was derived from the video lectures. The questions were on themes such as communication, focus under stress, teamwork and sleep deprivation (e.g. “Which of the following statements are true and which are not true?” “It is easier to recognise signs of sleep deprivation in yourself than in team mates. a. True. b. **False**”). The knowledge test had an internal consistency of α = 0.61. The test is available as Additional file 1.

#### Self-efficacy test

Existing self-efficacy tests are not sufficiently dedicated to patient safety. To assess self-efficacy regarding patient safety, a 12-item validated questionnaire on ability in communication issues (e.g. hand over patient information, perform debriefing) and on recognizing patient safety threats (e.g. recognition of sleep deprivation) was designed. Students rated their self-efficacy on a 1–100 scale (1 = *I cannot do this*, 100 = *I can do this perfectly*, see Additional file 2A). This approach has been validated for specific tasks in other studies [22]. The reliability of the self-efficacy scale (1–100) was good: α = 0.79. Factor-analysis showed that two constructs could be identified within the12-item scale: self-efficacy on *communication in patient safety issues* (e.g. ‘perform a debriefing’, 6 items, α = 0.81) and self-efficacy on *recognition of patient safety threats* (e.g. ‘recognize sleep deprivation’, 6 items, α = 0.79).

#### Questionnaire on perceived stress and patient safety awareness

In the absence of valid patient safety competencies assessment tools [23], a questionnaire was designed for perceived stress and awareness of patient safety issues and used during the 10-week clinical rotation. For perceived stress, the *Perceived Stress Scale,* a widely used validated psychological instrument for assessing the degree to which situations in one’s life are appraised as stressful was used as a basis [24]*.* We used three questions which were relevant in our context: how often students felt stressed, whether they felt they were able to cope with their tasks, and whether they felt to be in control. In addition, three questions on patient safety awareness were included: whether students had experienced any adverse events, what their own action in response to this event was and to provide an example of events and actions. The reliability of the 3-item reported stress scale (over 10 weeks) was high: 0.92. The reliability of the 2-item reported patient safety behavior scale (over 10-weeks) was good: 0.78.

All six questions related to the preceding week and were filled out on a weekly basis (including examples). Scales were rated from 1 (*never*) to 5 (*very often*). High scores indicated high levels of stress or adverse events, questions stated negatively were reversed before analysis (see Additional file 2B for the weekly questionnaire).

#### Evaluation questionnaire and interviews

Several questionnaires exist to evaluate (new) online instructional formats. For evaluation of the e-module and game, a combination was used of questionnaires on usefulness, ease-of-use, satisfaction, attitude, motivation [25, 26] and engagement [27, 28]. This resulted in a 23 item-questionnaire, with items scored between 1 (*strongly disagree*) to 5 (*strongly agree*, indicating positive opinions). Negatively stated questions were reversed before analysis. The questionnaire ended with an open question on positive and negative aspects of the game/e-module (see Additional file 2C). Factor-analysis showed that three constructs could be identified within the 23-item evaluation scale: the format is *educational,* i.e. stimulating to learn about patient safety (e.g. ‘the game/e-module helps me to be more effective at patient safety’, 9 items), *engaging* (e.g. ‘when playing the game/doing e-module, I felt actively involved’, 9 items), and *easy-to-use* (‘I quickly became skillful with the game/e-module’, 4 items). Reliability of scales was high to good (α = 0.93/ 0.92/ 0.74 regarding these scales, respectively). In addition to the items on evaluation, we added a question on how long they spent on the e-module or the game (“I have engaged in the game/read the e-module .. hours/min”).

In order to explore the strong and weak aspects of the game further, short semi-structured interviews have been conducted with seven game-players, starting with an open question on the value of the game and then going through the three parts. Important additional information from the interviews and open questions was summarized.

### Statistical analysis

Associations between categorical variables was assessed using chi-squared tests. ANOVA, post-hoc and independent sample *t*-tests were used to compare groups on continuous variables; paired t-tests were used to analyze differences within groups. Unless the distribution of scores was severely skewed, data from rating scales were analyzed as if they were interval without introducing bias [29]. Effect sizes (ES) were calculated using Glass’s delta [30]; ES ≥ 0.80 were considered large [31]*.* Associations were calculated using Pearson’s coefficient; we report coefficients ≥ 0.50. Confidence intervals of 95% were used to correct multiple correlations and for general analyses. A factor-analysis was performed on the self-efficacy and evaluation questionnaire, determining important constructs. The reliability of scales was assessed using Cronbach’s alpha. Missing data was treated with pairwise deletion; we used SPSS for the statistical analysis.

## Results

### Knowledge test

Test scores of the e-module group (M = 57.9, SD = 6.5) and game group (M = 60.1, SD = 6.7) were significantly higher than in the control group (M = 52.6, SD = 7.1, F(2,89) = 9.89, *p* < 0.0001), with large effect sizes (ES = 0.78 and ES = 1.11 for the e-module and game group resp.) There were no significant differences in scores between the game and e-module group.

### Self-efficacy

Mean scores on the two scales, communication in patient safety issues and recognition of patient safety threats, were low (12-item-scale, M = 67.1, SD = 9.2). Scores did not differ between groups on either scale. All students showed higher scores on the communication subscale (M = 68.8, SD = 10.8) than on the recognition of threats subscale (M = 65.4, SD = 12.7), t(99) = 2.30, *p* < 0.05).

### Reported stress and patient safety awareness

Average reported stress in the game group was lower than in the e-module group, but overall there were no significant differences between the three groups on stress or patient safety behavior and scores were low (Table 2). Most students (*n* = 86) reported examples of adverse events they had noticed during their rotation (e.g. medication related events) and in half of the cases (*n* = 38) they reported to have taken action in response (e.g. pointing out a possible drug interaction).

**Table 2:** Reported stress and patient safety behavior during a 10-week rotation per group (*n* = 68)

	Control group Mean (SD)	E-module group Mean (SD)	Game group Mean (SD)	P* (2-tailed)
Reported stress	2.2 (0.5)	2.4 (0.4)	2.1 (0.4)	0.2
Reported patient safety behavior	1.7 (0.5)	1.6 (0.4)	1.6 (0.6)	0.7

5 point scale, 5 = high level, 95% Confidence Interval. *Over 3 groups

### Learning time and game data

Self-reported average learning time for the game group was 2.9 h (SD = 1.1 h) and for the e-module group 0.9 h (SD = 1.0 h, *p* < 0.001). There was no association between learning time and outcome measures.

Due to the fact that the game did not yet have an “auto-save”-function, only log-data from 18 students was available. For this group, average game-time was 3.3 h (SD = 1.6 h). Logged game-time correlated with self-reported learning time (*r* = 0.77, *p* = 0.001). Students spent the majority of the game-time doing missions (M = 2 h., SD = 0.4 h.), followed by breathing exercises (M = 0.7 h., SD = 0.3 h.) and video-lectures (M = 0.7 h, SD = 0.4 h.). Students played the game on average 2.5 times. There was a significant association between knowledge scores and game-time (*r* = 0.56, *p* = 0.04), in particular between knowledge and video-lecture points (*r* = 0.68, *p* = 0.008). Game-time was also associated with patient-care mission points (*r* = 0.81, *p* < 0.001), indicating a learning effect within the game. Game-time and patient-care points correlated with self-efficacy (*r* = 0.51/ 0.50, *p* = 0.04) and negatively with reported stress during rotation (game-time – reported stress: *r* = − 0.65, *p* = 0.02; patient care points – reported stress: *r* = − 0.78, *p* = 0.002). This indicates that students who had successfully treated virtual patients and played longer were more self-confident in patient safety issues and experienced less stress during clinical practice, compared to students who were less successful and played shorter. There was no association between biofeedback or video-lecture points and reported stress, indicating an independent effect of the patient missions.

### Evaluation of the game and the e-module

**Fig. 3:**
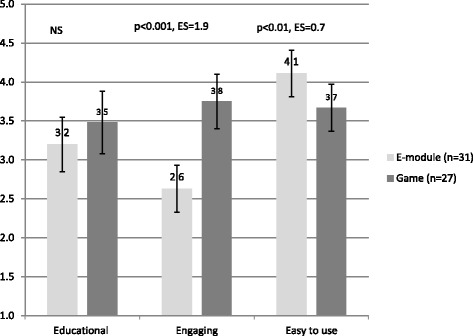
Evaluation of the e-module and game on three dimensions (ES = Effect Size)

The serious game was evaluated as more engaging than the e-module, with a very large effect size; the e-module was evaluated as more easy to use, with a medium effect size (Fig. 3). Although the game was evaluated as slightly more educational than the e-module, this difference was not statistically significant.

Most students responded to the request to name a few positive and/or negative aspects of the e-module or game. In summary, the game was perceived as ‘fun to do’, with interesting topics; in particular the patient-missions were appreciated for training in decision making, offering challenge, creating awareness, and learning to deal with stress (“time flies”, “the missions generate stress so you become more aware of what is coming”). The video-lectures were evaluated as interesting, but it took too long before the player could go to the fun part (the missions), and after a while they became bored (“hard to keep concentration”, “not interactive”). The breathing exercises were evaluated as useful but less realistic, they often took too long and did not always work well (“exercises do not match reality”). The e-module was evaluated as dealing with important topics and of educational value, but boring and with too little interaction (“too much text on one page”, “not interactive”, “no examples”). The interviews confirmed that the missions were the most motivating part: educational and realistic, with an attractive storyline. One particularly well-appreciated part of the game was the ‘red flag game’: an interactive exercise with clinical situations depicting ‘red flags’ in communication and team performance that should be recognized as specific threats to patient safety (with a score and feedback). Several students mentioned that this is a good format and they would remember the messages.

## Discussion

Fourth-year medical students were randomly allocated to extra educational patient safety content either in the form of a serious game or an e-learning module. Compared to students in a historic control group who had received no additional instruction, patient safety knowledge improved both after studying with the game and the text-based e-module, with large effect sizes. During the following rotation, students from all three groups reported low levels of stress, patient safety awareness and self-efficacy, with no differences between groups.

Our first hypothesis that patient safety knowledge would improve using video-lectures (in the game) or text-based lectures (in the e-module), compared to no extra education in the control group was confirmed. Video-lectures from experts can be inspiring, but text is more flexible to read and can be adjusted to the readers’ interests. This is consistent with results from media-research indicating that information can be presented in a variety of media with equal learning outcomes, but at very different costs and access [32]. In addition, the effectiveness of online instruction for patient safety knowledge and attitudes is also confirmed [33]. Students who spent more time on the video-lectures in the game demonstrated more patient safety knowledge, but this may be a spurious relationship. The game group spent considerably more time on learning: three hours versus one hour in the e-module group.

Our second hypothesis, i.e. that self-efficacy and patient safety awareness would be higher in the game group than in the e-module or control group, was not confirmed. The low self-efficacy levels of all students before their first rotation can possibly be explained by the absence of clinical experience; it is hard for students to estimate how they will be able to deal with stress and communication. In another study on the same game, students did the self-efficacy test before and after engaging with the game and did show improved scores on most items of the test [19]. However, this may be the result of the fact that students feel they should be more confident on patient safety issues after having played a game on this subject [19]. A randomized posttest-only design with a control group (as used in the current study) is less affected by these validity threats [34, 35]. Although students from the game group spent most of their gaming-time treating virtual patients and doing exercises on communication and teamwork, developing patient safety awareness and changing behavior are complex and ambitious as learning goals. A systematic review on patient safety education showed only a few reports of positive outcomes on higher-level skills and attitudes [14, 16]. More knowledge on effective educational designs for developing awareness and assertive behavior is needed. The observation that our group of students did not benefit from the patient-missions does not mean that the game may not be effective for other groups, for example residents. In an earlier study on the effectiveness of a case-based simulation game on emergency care skills we found that although the game was effective for residents, it was too difficult for students and only residents showed improved skills [36].

Our third hypothesis that perceived stress levels during rotation would be lower in the game group (due to the biofeedback exercises) than in the other groups was not sustained either. A possible explanation for this finding is the low level of experienced stress by all students, who are at a very early career stage and have not yet experienced the burden of clinical responsibility. Students sometimes reported stress during rotations, but this appeared to be more related to how they felt they must present themselves to medical staff [37]. As a result of these low levels of baseline stress, we do not know from this study whether the exercises in the game are effective for stress reduction or not. It would be interesting to perform a follow-up study with first-year residents who have more responsibility for patient care and possibly related stress. A comparison could be made between the biofeedback exercises in the game and an audio-based mindfulness training which has shown to be effective in reducing stress levels in medical students [38]. Interestingly, students who had treated patients successfully during game-missions, reported higher levels of self-efficacy and experienced less stress during rotations than students who were less successful in the game. However, since the game group as a whole did not show better self-efficacy or less stress, this may be a spurious relationship (more motivated students played longer and were more self-confident, unrelated to the intervention).

Our fourth hypothesis that students in the game group would be more motivated to learn was confirmed. Students evaluated the serious game as considerably more engaging than the e-module and also spent considerably more self-study time (3 h on the game and 1 h on the e-module). In particular the patient-missions and the exercises in patient safety situations were positively evaluated. Considering the fact that the e-module lacked any type of patient missions, we can only compare the instructional formats in a limited way regarding engagement. We can conclude the game worked well to stimulate students’ engagement, even though it was more time consuming than the e-module. In general a majority of medical students, including many who do not regularly play video games, hold favorable views on its use in medical education to experience different clinical situations [39]. The e-module was evaluated as more easy-to-use, which is understandable from the simplicity of its format.

To the best of our knowledge, this is one of the first studies on the comparative effectiveness of two modern digital formats on performance outcomes and motivation in health professions education. Yet, our study has several limitations.

This study was done with 4^th^-year medical students, who have limited clinical responsibilities during their rotations. Non-technical skills such as recognizing and dealing with stress and communicating well during difficult circumstances are central learning goals of the game and were designed with the ‘overwhelmed’ young medical resident in mind. These issues are less relevant to medical students making their first entry into the clinic. Our results cannot be generalized to other groups and may therefore be different for residents. Nonetheless we decided to first compare the effects of the game and an e-module in medical students, because it is hard to motivate busy medical residents to spend extra study time on new educational formats and tests without evidence for their effectiveness. Moreover, the results of this study may serve as a baseline-study for future studies with residents.

A second limitation is the risk of confounding, as the content and learning time of the two online interventions was not the same: the e-module lacked patient missions (aimed at patient safety awareness and effective communication). This may to some degree explain the differences in engagement between the game and e-module. Games are formats with often unique and intertwined features, for which an equivalent is not easy to develop in another format. This is a common issue in research on computer-based learning [40]. We tried to address the possible confounding effect by clearly describing the separate components of the interventions with its learning goals. There was little overlap in the components and its learning goals, as a result we were able to make deliberate assumptions on which component contributed to a specific learning effect (e.g. the video lectures contributed to patient safety knowledge). As we did not find any differences between groups in patient safety awareness and effective communication, we do not believe confounding influenced our conclusions. However we recommend in future studies to design a more rigorous controlled intervention design.

A third limitation is the fact that this game consisted of different components, and it is unclear which component actually contributed to the differences found, e.g. in the evaluation of the game and e-module (the game was perceived as more engaging and less easy-to-use compared to the e-module). It was clear from the interviews that the most engaging part were the patient missions, but in a follow-up study we recommend these components should be evaluated separately, e.g. comparing the e-module alone with an e-module and patient missions condition, preferably with residents.

A fourth limitation is that as historical comparison was used for the control group, we did not have control over historical changes in education. Patient safety education in the medical curriculum is in constant flux. During data-collection for the control group, the focus in patient safety education was on the human-factor approach; during data-collection for the intervention groups, it was more focused on effective and open communication in general. This may have caused a greater patient safety awareness among the control group, implying this higher ‘baseline’ may have decreased the difference in patient safety awareness compared with the intervention groups. Results might have differed if the control group was included in the second phase of the study, but considering the low mean scores and low variance for all three groups, we do not believe this has influenced our conclusions (which are mainly related to a comparison between the game and e-module group).

Finally, we only measured self-perceived performance in clinical practice. Although it is difficult to assess patient safety behavior in clinical practice and students reported in detail on adverse events they perceived and their personal responses to them, in a follow-up study actual patient safety behavior in each group should be assessed.

## Conclusions

This study showed that video-lectures from a serious game and text-based lectures from an e-module are equally effective in developing knowledge on specific topics such as patient safety. The game was evaluated as more engaging than the e-module; the e-module was evaluated as more easy to use than the game. Although this serious game, in which students were able to practice their skills in simulated scenarios, was strongly engaging for them and stimulated them to study longer, it did not result in improved performance. More research is needed into the effects of game-design features on learning outcomes.

## Additional file

Additional file 1: Knowledge test. (DOCX 38 kb)
Additional file 2: (ZIP 1170 kb)
