# Supplementary material for: Comparative effectiveness of a serious game and an e-module to support patient safety knowledge and awareness
Source: BMC Med Educ. 2017 Feb 2;17:30. doi: 10.1186/s12909-016-0836-5 (PMC5289006; doi:10.1186/s12909-016-0836-5)
Supplement: Additional file 1: — Knowledge test. (DOCX 38 kb) [file 12909_2016_836_MOESM1_ESM.docx]

**Knowledge test**

**Patient safety**

**70 items; Correct answers in bold. MA= multiple answer questions.**

**Communication**

*Pre-Briefing*

1.Which of the following components belong to a complete patient safety pre-briefing? **MA**

1. A short description of the situation Yes / **no**
2. A summary Yes / **no**
3. Acknowledgement of all communication **Yes** / no
4. An invitation for participation **Yes** / no
5. The mechanism of injury Yes / **no**
6. Introducing yourself **Yes** / no
7. A motivational message Yes / **no**
8. Questions to ensure understanding **Yes** / no
9. A description of the task Yes / **no**
10. A checklist consisting the expected outcomes **Yes** / no

2. Pre-briefing is particularly important before

1. surgical procedure
2. administration of medication
3. **any activity involving teamwork**
4. handing over a patient

3. A team huddle is:

1. a group debriefing
2. **a pre-briefing**
3. a strategy meeting during action
4. a post-hoc meeting to reconcile a dispute

4. Which of the following skills belong to the ‘showing good interpersonal skills’ component of a pre-briefing? **MA**

1. Showing empathy Yes / **no**
2. Introducing yourself  **Yes** / no
3. Making eye-contact **Yes** / no
4. Energizing the team Yes / **no**
5. Acknowledging all communications Yes / **no**
6. Getting to know all team members names  **Yes** / no
7. Using adequate intonation while communicating Yes / **no**

5. Which item is not important during the communication of a message?

a. Body language

b. **The order of words**

c. Word choice

d. Pronunciation of words

*Assertive Statement*

‘Dr Jones, don’t you think we should treat this patient for his high bloodpressure?’ is an example of an incomplete “effective assertive statement”. Check which components are included, which are missing and which are not essential for an effective assertive statement.

1. Get the attention **included** / missing / not essential
2. Show your concern included / **missing** / not essential
3. Ask for help included / missing / **not essential**
4. State the problem included / **missing** / not essential
5. Wait for a solution included / missing / **not essential**
6. Propose a solution **included** / missing / not essential
7. Wait until the problem has been solved included / missing / **not essential**
8. Wait until a decision has been made included / **missing** / not essential

*Call Back Read Back*

14. A specialist asks you to give one 1ml ampulla morphine 10mg/ml. You reply: ‘here is one 1ml ampulla morphine 10mg/ml’ and you hand over the medication. This is an example of:

1. ‘Pre-briefing’
2. **‘Call back-read back’ principle**
3. ‘Debriefing’
4. ‘Hands off eyes on’ principle
5. The ‘comprehension check’

15.The ‘call back read back’ principle teaches to always check the results of your previous ordered diagnostic tests.

1. True
2. **False**

16. The ambulance brings in an ill patient while you are on duty in the Emergency Room as a medical officer. You read the admission papers of the patient, which have been handed over by the ambulance nurse.

This is an example of the ‘read-back’ part of the ‘call back-read back’ principle.

1. True
2. **False**

17. In a community hospital the statement ‘Communication that goes unacknowledged did not happen’ is:

1. **True**
2. False

*Handovers*

18. One appropriate way of conducting a handover is debriefing.

This statement is:

1. True
2. **False**

19. Part of a handover procedure is the ‘call back-read back’ action.

This statement is:

1. True
2. F**alse**

20.Do the following components belong to a complete handover? **MA**

1. A notation **Yes /** no
2. A summary Yes / **no**
3. A debriefing Yes / **no**
4. Information provided according an agreed structure **Yes /** no
5. Supplying a written summary Yes / **no**
6. Providing a differential diagnosis Yes / **no**
7. A dialogue  **Yes /** no
8. A therapeutic plan Yes / **no**

21. The ‘Hands off-eyes on principle’ applies to

1. Electric cardioversion
2. **Handovers**
3. Pre-briefing
4. Handling conflicts
5. Call back Read back

22. The ‘Comprehension check’ should be carried out after:

a. Call back Read back

b. Hands off-eyes on principle

c. Debriefing

**d. Handovers**

23. Which of the following steps belong to a complete debriefing and put them in the right order:

A. Checking for all unanswered questions/unaddressed problems

B. Accurate description of what happened

- 1. Things that need improvement
  2. Specific action for changing or improving next performance
  3. Thorough description of why we did what we chose to do
  4. Things that went well

**Correct order: B, E, D**

24. A synonym for debriefing is a performance feedback session

1. **True**
2. False

25. Debriefing is important because:

1. it makes patient handover safer
2. it clarifies what all team members tasks are
3. it is a check for unanswered questions
4. **its effect is continuous quality improvement**

26. The goal of a debriefing is:

1. to optimise the final preparation for a task
2. **to optimise the performance for the next task**
3. to make sure the task is completed totally

**Focus under Stress**

*Focus on your Patient*

*Focus on the Now-through centred breathing*

27. “Centred breathing” is a

- 1. relax aimed breathing technique
  2. 5-step breathing technique
  3. **focus directed breathing technique**
  4. mid-abdominal breathing technique

***Teamwork***

*Shared mental model*

20.Which of the following statements about ‘the shared mental model’ are true and which are false? The shared mental model:

28. helps to detect possible mistakes of teammates

**a. True**

1. False

29.is based on clear communication between teammates

1. **True**
2. False

30.aims at intuitively knowing what teammates are doing

1. True
2. **False**

31.prevents depression in team mates

1. True
2. **False**

32.shows the distribution of stress applied to a team by external pressure

1. True
2. **False**

33.is a standard approach that every teammate knows about

1. True
2. **False**

34.spreads the effects of sleep deprivation among team mates

1. True
2. **False**

*Teamwork: Ken Catchpole –teamwork to control stress level*

*Using Resources*

Which of the following sentences are true and which are false?

The use of resources in dealing with patient safety issues is about:

1. reducing the stress of one team member to other members
   1. True
   2. **False**
2. knowing and using your equipment
3. True
4. **False**
5. using the knowledge and skills of other team members
6. **True**
7. False
8. asking a colleague for help
9. **True**
10. False

**Sleep Deprivation**

*Signs of Sleep Deprivation*

Which of the following statements are true and which are not true*?*

39. Sleep deprivation is associated with a significant increase in medical mistakes.

1. **True**
2. False

40. It is easier to recognise signs of sleep deprivation in yourself than in team mates

1. True
2. **False**

41. The ‘call back-read back’ principle is one of the best methods to prevent mistakes due to sleep deprivation.

1. True
2. **False**

42. Sleep inertia is the phenomenon that you feel really tired the first couple of minutes after a short nap

**a. True**

b. False

43. The best method to deal with sleep inertia is to adapt to the local day-night pattern

a. True

**b. False**

44. To prevent sleep deprivation it is best to take an hour more sleep every day for 4-7 days.

a. True

**b. False**

45. The recommended time for a short nap is 10 to 15 minutes

**a. True**

b. False

46. Short naps do not make one more alert in the first half hour later

**a. True**

b. False

47. Self-awareness is impaired by sleep depriviation

**a. True**

b. False

48. When people ignore their feelings of tiredness, they can be able to get over their sleepy feelings and function normally.

1. True
2. **False**

49. Self awareness is more related to self-confidence than to unsecurity.

1. True
2. **False**

**Depression**

*Signs of Depression*

50. Physicians are better skilled in recognising signs of depression in themselves than non-physicians.

1. True
2. **False**

51. Which of the following statements belong to a sign of depression? **MA**

1. Insomnia or hypersomnia  **Yes /** no
2. Feeling hopeless **Yes /** no
3. Having a flight of ideas Yes / **no**
4. Changes in weight **Yes /** no
5. Feelings of hurting yourself or others **Yes /** no
6. Feeling more sad in the evening Yes / **no**

52. Working as a resident increases the risk of depression.

This statement is:

**a. True**

b. False

53. When you think a colleague is depressed, you should talk to a person of authority about it.

This statement is:

**a. True**

b. False

54. You should ask friends and family to pay attention to possible signs of depression.

This statement is:

**a. True**

b. False

*Depression in Teammates*

*Depression & Drug Abuse*

55. If you recognise signs of alcohol or drug abuse in fellow residents, you should talk with them about your suspicion.

1. true, because it is so easy for physicians to obtain narcotic drugs
2. **true, because it is hard to recognise in oneself**
3. false, because this only feeds feelings of distrust among residents
4. false, because this is a person’s own responsibility

56. Alcohol or drug abuse can mask a depression; this is its major threat to patient safety.

This statement is:

1. True
2. **False**

57. Working as a resident increases the risk of alcohol or drug abuse

This statement is:

- 1. **True**
  2. False

*Making Mistakes when depressed*

58. Residents showing evident signs of depression make about 10 times more errors then colleagues without such signs

1. this number is substantially higher
2. **this number is substantially lower, but still higher than in non-depressed colleagues**
3. it has not been documented that they make more mistakes

59. The 4 most probable reasons for making more mistakes while being depressed are: **MA**

1. **a slower reaction time**
2. feelings of depression
3. **a lack of motivation**
4. making less eye-contact in communication
5. being very tired the first 10-15 minutes after a nap
6. **increased forgetfulness**
7. **feelings of disassociation**
8. thoughts of hurting oneself or other people

*Patient Safety*

*Introduction to Patient Safety*

60. Patients admitted to hospital encounter unintended harm due to adverse events during their stay. The correct percentage is:

1. 1-3%
2. **3-6%**
3. 6-12%
4. 12-20%
5. 20-30%

61. Unintended adverse events in patients during hospitalization are a more common cause of death in the USA than car accidents.

This statement is:

1. **True**
2. False

62. The majority of unintended adverse events in patients during hospitalization is due to:

1. a lack of motivation in the medical staff to observe patient safety rules
2. a lack of knowledge and/or skills medical staff about patient safety
3. **system problems of the hospital in dealing with patient safety**

63. The Systems Engineering Initiative for Patient Safety (SEIPS) work system model is an excellent tool to evaluate a working environment.

This statement is:

**a. True**

b. False

64. Which item(s) belong(s) to an element of the SEIPS work system model? **MA**

1. 91. The tools and technology **Yes** / no
2. The working team Yes / **no**
3. The individual **Yes /** no
4. The training and education Yes / **no**
5. The task **Yes /** no
6. The organisation **Yes /** no

*The 4 challenges of Patient Safety*

65. Do the following statements belong to the challenges of patient safety? **MA**

1. High level of ambiguity  **Yes /** no
2. High level of carelessness Yes **/ no**
3. Problems are hardly visible **Yes /** no
4. High level of complexity  **Yes /** no
5. High level of indifference Yes **/ no**
6. Conflicting interests Yes **/ no**
7. The problem is linked to several side-effects of professionalism **Yes /** no

66. The high level of complexity is one of major challenges in:

1. **Patient safety matters**
2. Sleep deprivation
3. Identifying red flags in teamwork
4. Providing a good handover

67. A high level of ambiguity can mean:

- 1. **The discussion of system failure versus individual failure**
  2. Hierarchical pressure
  3. Peer pressure
  4. Difficulties asking colleagues

*9 Red Flags*

68.Which are the nine red flags and which are not? **MA**

1. Conflicting inputs **Yes /** no
2. Ambiguity Yes / **no**
3. Preoccupation  **Yes /** no
4. Lazyness Yes / **no**
5. Imprudence Yes / **no**
6. Complex situations Yes / **no**
7. Not communicating  **Yes /** no
8. Confusion **Yes /** no
9. Rudeness Yes / **no**
10. Violating policy or procedure **Yes /** no
11. Failure to meet a target **Yes /** no
12. Prematurity Yes / **no**
13. Inaccuracy Yes / **no**
14. Indifference about a situation Yes / **no**
15. Not addressing a discrepancy  **Yes /** no
16. Fatigue  **Yes /** no
17. Carelessness Yes / **no**
18. Indiscretion Yes / **no**
19. Stress  **Yes /** no

69. The red flags are warning signs of:

1. Depression
2. Sleep Deprivation
3. **Teamwork**
4. Patient status

70. A red flag should prompt all attending personal to initiate advanced live support.

This statement is:

1. True
2. **False**
