# Supplementary material for: Comparative effectiveness of a serious game and an e-module to support patient safety knowledge and awareness
Source: BMC Med Educ. 2017 Feb 2;17:30. doi: 10.1186/s12909-016-0836-5 (PMC5289006; doi:10.1186/s12909-016-0836-5)
Supplement: Additional file 2: — (ZIP 1170 kb) [file 12909_2016_836_MOESM2_ESM.zip › Appendix/App A_Self-efficacy.pdf]

Questionnaire Self-Efficacy

100 mm visual analogue scale self-efficacy questions
"Please indicate how you assess your ability to …

1 Physically reduce high levels of stress in yourself	1-100
2 Focus on one important task when dealing with multiple things at a time	1-100
3 Perform a debriefing within a team, e.g. before an operation	1-100
4 Hand over patient information	1-100
5 Recognize signals of threats to patient safety during teamwork	1-100
6 Conduct a debriefing after a team task	1-100
7 Raise issues of threat to safety of a patient among the medical staff	1-100
8 Recognize signs of depression in yourself	1-100
9 Recognize signs of depression in colleagues	1-100
10 Recognize signs of sleep deprivation in yourself	1-100
11 Recognize signs of sleep deprivation in colleagues	1-100
12 Approach a senior staff member personally for his or her negligence	1-100
