# Supplementary material for: Comparative effectiveness of a serious game and an e-module to support patient safety knowledge and awareness
Source: BMC Med Educ. 2017 Feb 2;17:30. doi: 10.1186/s12909-016-0836-5 (PMC5289006; doi:10.1186/s12909-016-0836-5)
Supplement: Additional file 2: — (ZIP 1170 kb) [file 12909_2016_836_MOESM2_ESM.zip › Appendix/App C_Evaluation Questionnaire.pdf]

Evaluation questionnaire

1. How experienced are you in electronic games such as gaming apps on your mobile, games for the pc, consoles etc. ?

   0 - NONE: I have no experience in gaming. I do not play games at all.
   1 - BASIC: I have some basic gaming experiences, I play 0– 2 hours per week.
   2 - MODERATE: I have basic gaming experiences. I play 2-4 hours a week .
   3 - INTERMEDIATE: I consider myself experienced. I play 4-6 hours a week.
   4 - EXPERT: I consider myself being an expert in gaming. I play 6+ hours a week.

2. I played AMS-1 until the end: YES/NO
   If ''no'', where did you stop and why? …….

3. I have played AMS-1 : …..hours…..minutes

4. During the Education Week , did you allow access to the e-module or AMS1 to someone participating in the research who was not officially allowed access?
   ....

		Strongly disagree	disagree	Neutral	Agree	Strongly agree
	**Usefulness**					
1	AMS-1 helps me to be more effective at Patient Safety					
2						
3	AMS-1 is useful for Patient Safety education					
4	AMS-1 gives me control on Patient Safety activities during my clerkship					
5	AMS-1 meets my needs					
	**Ease of use**					
6	AMS-1 is easy to use					
7	Learning to operate AMS-1 initially is full of problems					
8	I easily remember how to use AMS-1					
9	I quickly became skilful with AMS-1					
	**Satisfaction**					
10	I am satisfied with AMS-1					
11	I would recommend AMS-1 to fellow students					
12	AMS-1 is fun to use					
13	AMS-1 is pleasant to use					

	Engagement					
14	When playing AMS-1, my attention was entirely on the game					
16	When playing AMS-1 I felt actively involved in the game					
17	I could concentrate fully throughout the game					
18	The content of the game is educational					
19	I play AMS-1 without thinking about how to play					
	**Attitude towards using**					
20	It is a good idea to use the AMS-1 for my study					
21	I like the idea of using a game (AMS-1) to learn about patient safety topics					
22	Overall, I enjoyed using AMS-1					
	**Behavioural Intention to Use**					
23	I would have liked to continue playing AMS-1					
	**Motivation**					
24	It is important for medical students to learn about patient safety topics					
25	I like what we are learning in this game					
26	I can use the things we learn in this game in my future work as a doctor					
27	I try to learn more about patient safety to be a better doctor in the future					

List the most **negative** aspect(s):

1.
2.
3.

List the most **positive** aspect(s):

1.
2.
3.
