# Supplementary material for: Comparative effectiveness of a serious game and an e-module to support patient safety knowledge and awareness
Source: BMC Med Educ. 2017 Feb 2;17:30. doi: 10.1186/s12909-016-0836-5 (PMC5289006; doi:10.1186/s12909-016-0836-5)
Supplement: Additional file 2: — (ZIP 1170 kb) [file 12909_2016_836_MOESM2_ESM.zip › Appendix/Appp B_Awareness en Perc stress.pdf]

Questionnaire on perceived stress and patient safety awareness (weekly)

Perceived Stress Scale questionnaire

0 = Never 1 = Almost Never 2 = Sometimes 3 = Fairly Often 4 = Very Often

0 1 2 3 4

1. During the last week, how often have you felt nervous and “stressed” in your clerkship?
2. During the last week, how often have you found that you could not cope with all the things that you had to do during clerkship?
3. During the last week, have you felt you were in control during your clerkship?

Patient Safety

The next three questions are related to 'adverse events'. An ‘adverse event’ is a (possibly) negative outcome resulting from a medical intervention, which is not due to the underlying condition of the patient. Examples are violations of rules, near misses and mistakes.

0 1 2 3 4

4. Have you seen/experienced any adverse events during the last week?
5. Have you taken any action in response to this event/ these events?
6. (if question 8 was answered affirmatively): Can you give an example of such action?
